# Use of Self-Assembled Colloidal Prodrug Nanoparticles for Controlled Drug Delivery of Anticancer, Antifibrotic and Antibacterial Mitomycin

**DOI:** 10.3390/ijms23126807

**Published:** 2022-06-18

**Authors:** Mohamed M. Abdelghafour, Ágota Deák, Diána Szabó, Imre Dékány, László Rovó, László Janovák

**Affiliations:** 1 Department of Physical Chemistry and Materials Science, University of Szeged, Rerrich Béla tér 1, H-6720 Szeged, Hungary; m.abdelghafour2015@yahoo.com (M.M.A.); dagota13@yahoo.com (Á.D.); i.dekany@chem.u-szeged.hu (I.D.); 2 Department of Chemistry, Faculty of Science, Zagazig University, Zagazig 44519, Egypt; 3 Department of Oto-Rhino-Laryngology and Head & Neck Surgery, University of Szeged, Tisza Lajos krt. 111, H-6724 Szeged, Hungary; diniklinik@freemail.hu (D.S.); office.orl@med.u-szeged.hu (L.R.)

**Keywords:** modified PVA, Mitomycin, self-assembled polymeric particle formation, mucoadhesive properties, prolonging/adjusted drug release

## Abstract

Herein we present the synthesis of a polymeric prodrug nanomaterial capable of spontaneous, self-assembled nanoparticle formation and of the conjugation (encapsulation) of drugs with amino and/or carboxyl and/or hydroxyl groups via ester and/or amide linkage. Mitomycin C (MMC) a versatile drug with antibiotic, antibacterial and antineoplastic properties, was used to prove this concept. The in vitro drug release experiments showed a fast release for the pure MMC (k = 49.59 h^−n^); however, a significantly lower MMC dissolution rate (k = 2.25, 1.46, and 1.35 h^−n^) was obtained for the nanoparticles with increased cross-link density (3, 10, 21%). The successful modification and conjugation reactions were confirmed using FTIR and EDX measurements, while the mucoadhesive properties of the self-assembled particles synthesized in a simple one-pot reaction were proved by rheological measurement. The prepared biocompatible polymeric prodrugs are very promising and applicable as a drug delivery system (DDS) and useful in the area of cancer treatment.

## 1. Introduction

Orally administrated Mitomycin C (MMC) has long been used as an antibiotic, antifibrotic and antineoplastic agent, and the antitumor mechanism of MMC is based on its use as a bifunctional alkylating agent that inhibits DNA synthesis [1]. Still, its undesirable side effects (such as renal damage, gastrointestinal damage, and bone marrow depression [2,3]) minimize the dose in chemotherapy. However, around eight times the dosage is required compared to intravenous (IV) and intraperitoneal (IP) administration; in addition, oral administration of MMC is 3 to 12 times the injection administration of LD_50_ [4]. Therefore, it is necessary to develop prodrugs or so-called drug delivery systems (DDS) to provide the prolonged therapeutic effect and safe administration of MMC to reduce its side effects and enhance therapeutic efficiency.

Nano- or microparticulate encapsulation of MMC for controlled release has benefits including lower dosage, decreased side effects, enhanced bioavailability, high carrier capacity, high stability, and better treatment performance. The prevalent method of delivery of MMC includes drug encapsulation in nano- or micro-particles such as dextran [5,6], albumin [7], poly butyl cyanoacrylate [8], estradiol [9], hydrogels [10], and poly-epsilon-caprolactone [11]. However, these systems have demonstrated some shortcomings in the local administration of MMC as they rapidly degrade in the body.

A polymeric prodrug is the conjugation of a drug with a polymer to form an inactive precursor of a drug. According to Ringsdorf’s model [12], a polymeric prodrug mainly contains five components: polymeric backbone, spacer, drug, soluble agent, and targeting group. Polymeric prodrugs have several advantages, including prolongation of drug action, controlled drug release, and immuno-protection during cancer treatment. The coupling of the drug with a polymer via an amide bond is strongly promising because of its high stability against different reaction conditions and high temperatures [13]. In this scenario, a comparatively significant amount of prodrug can be administered without attendant side effects and overdosing risk.

Mucoadhesion is an attachment phenomenon of the natural or synthetic polymer to the surface of the mucosa. Mucoadhesive drug delivery systems (MDDS) may be configured to specifically target oral, rectal, buccal, vaginal, ocular, and nasal routes of administration [14]. Thiolated polymers are the most effective mucoadhesive polymers because they can easily form covalent bonds (disulfide bonds) with cysteine-rich sub-domains of the mucus gel layer, in addition to the hydrogen bonds and van der Waals’ forces that lead to an increase in the drug residence time and improve bioavailability [15,16].

Polyvinyl alcohol (PVA) provides a suitable base for DDS because of its good mechanical properties, biodegradability, biocompatibility, and ease of modification [17,18]. The biocompatible PVA has FDA approval for in vivo applications and clinical uses in the human body [19,20,21]. Baker et al. revealed that preclinical and clinical studies of PVA validated its safety and biocompatibility without any adverse effects, indications of tissue loss, or cytotoxicity [19]. PVA with pendant OH groups can be converted into carboxylic acid derivatives by treatment with acid anhydrides (such as succinic anhydride). For drug and protein conjugation, the succinyl group integrated into the polymer can act as a spacer between the drug and the polymer, which controls the rate release through enzymatic and/or hydrolytic (non-enzymatic) cleavage of the temporary bond between the drug and polymer. In the in vitro environment (enzyme-free condition) via an aqueous buffer solution, the rate of hydrolysis of the temporary bond (e.g., amide or ester bond) may be too slow and not therapeutically effective. However, amidase or esterase in an in vivo environment will result in a significant catalytic acceleration of hydrolysis kinetics from twofold to multiple magnitude orders [22]. In addition to the conjugation of the drug, the succinyl group provides suitable cross-linking groups (COOH) with the OH groups of the PVA backbone by the formation of an ester bond via the use of 1-(3-Dimethylaminopropyl)-3-ethylcarbodiimide hydrochloride (EDC) as a coupling agent, according to reported literature [23], resulting in the formation of self-particles during the conjugation reaction of MMC to succinated PVA.

In the present work, we aimed to reduce the severe side effect of MMC by synthesizing a polymeric prodrug and increasing the mucoadhesive properties of the prepared polymeric prodrug by conjugation of an aminothiol compound (e.g., cysteamine, CYS) to increase the terminal free thiol group that can form a disulfide bond with the mucous membrane. The in vitro release experiments were performed for 7 days under physiological conditions (PBS, pH = 7.4) with the application of different kinetic models to evaluate the releasing kinetics (zero-order, first-order, Hixson–Crowell, Higuchi, and Korsmeyer–Peppas models) [24]. The relevant antibacterial and anticancer properties of the obtained polymeric prodrugs were examined. The prepared conjugated particles with strong mucoadhesive properties were suitable for the prolonged drug release of MMC under physiological conditions. The release of MMC could be controlled by adjusting the cross-linking density.

## 2. Results

### 2.1. Structural Characterization of the Synthesized Samples

**Figure 1:**
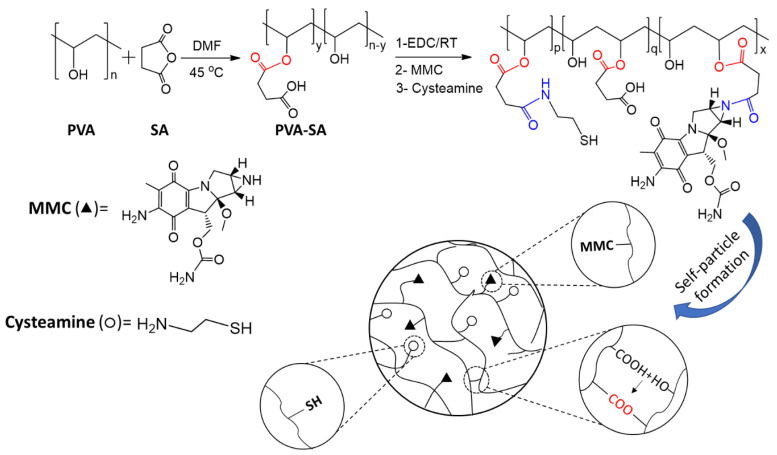
The scheme of the succinylation reaction of PVA and conjugation reaction of MMC and cysteamine (through amide bonds highlighted in blue) with PVA-SA in addition to the self-particle formation by cross-linking of the OH group and COOH group by EDC (through ester bonds highlighted by red).

As shown in Figure 1, the initial PVA was functionalized with various molar ratios (from 8 to 126 molar %) of succinic anhydride to the PVA’s OH groups (15.8 mmol/g, determined by acetic anhydride/pyridine titration [25]).

The substitution degree of succinate moiety ranged from 3.1 to 21.5% (see Figure S1B in the Supplementary Materials). Due to the mild reaction condition and the applied DMF as a poor solvent for PVA, the percent of substitution was relatively low because the reaction only occurred on the surface of partially dissolved PVA particles, however, using extreme conditions for substitution reaction will lead to the crosslinking of PVA by SA which was not preferred for our work as was studied by Zhou et al. [26]. The attachment of the carboxyl group to the PVA backbone and the partial carboxylation (PVA-SA) provided a suitable candidate polymer for conjugation reaction using EDC as a coupling agent. Next, the obtained PVA-SA was reacted with cysteamine (CYS) and MMC via amide bond formation, with the MMC binding range between 70 and 80%. Furthermore, the remaining OH and COOH groups allowed the self-particle formation of linear polymer chains through ester bonds (Figure 1) [23].

**Figure 2:**
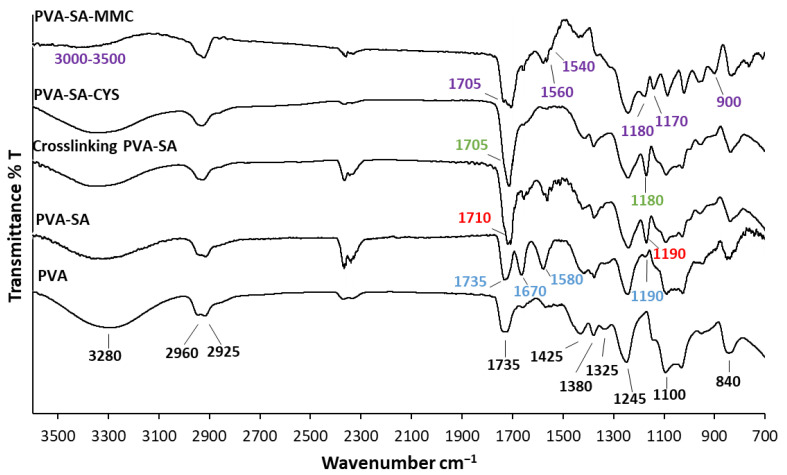
FTIR spectra of initial PVA and modified forms.

The successful reactions were confirmed using FTIR measurements as presented in (Figure 2).

The main characteristic peaks of PVA were 3280 cm^−1^ (O–H stretching vibration), 2960 cm^–1^ (CH_2_ asymmetric stretching vibration), 2925 cm^−1^ (CH_2_ symmetric stretching vibration), 1735 cm^−1^ (C=O carbonyl stretch), 1425 cm^−1^ (C–H bending vibration of CH_2_), 1380 cm^−1^ (C–H deformation vibration), 1325 cm^−1^ (CH_2_ wagging vibration), 1245 cm^−1^ (C–O–C stretching vibration), 1100 cm^−1^ (C–O stretching of acetyl groups), and 840 cm^−1^ (C−C stretching vibration) [25,27,28]. It was obvious that when the OH groups of the PVA backbone were modified by succinyl moieties, the OH-stretching band of pure PVA (~3280 cm^−1^) diminished, and the emergence of significant absorption bands at 1735 and 1670 cm^−1^, which were associated with the esteric carbonyls and carboxylate moieties, respectively, characterized the difference between these two spectra [29]. The new peak at 1580 cm^−1^ was due to the asymmetric stretching vibration of carboxylate RCOO^−^ [30]. The new absorption band between 1150 and 1190 cm^−1^ for the PVA-SA arose from the C–O stretching vibrations of the ester group [31]. This was evidence that the successful introduction of succinic moiety into the PVA side chains occurred. The conjugation reaction of PVA-SA with CYS was proved by the vanishing of the band at 1670 and 1580 cm^−1^ related to carboxylate moiety and asymmetric stretching vibration of carboxylate RCOO^−^, as well as the shift and increasing the intensity of the carbonyl band (C=O) at 1710 cm^−1^ owing to the formation of an amide bond between the carboxylate group of PVA-SA and the NH_2_ group of cysteamine. Moreover, the emergence of a new peak at 1180 cm^−1^ was due to the C−N stretching vibration, which confirmed the successful occurrence of the conjugation reaction.

The disappearance of the NH band at 3442 cm^−1^ and the appearance of the amide band (C=O) at 1705 cm^−1^ meant that the conjugation reaction successfully occurred between PVA-SA and MMC. In addition, there was a change in the region from 3000 to 3500 cm^−1^ in the case of the conjugated products, reducing the peak at 1670 cm^−1^. This was attributed to the carboxylate moieties and proved that the reaction occurred between the NH of MMC and the carboxyl group of PVA-SA. MMC moiety was characterized by the emergence of new peaks at 2850, 1383, 1560, 1540, 1170, and 900 cm^−1^ [32].

Spontaneous particle formation by cross-linking between the simultaneously available OH and COOH groups was confirmed by decreasing the band that related to the carboxylate moiety of succinate and the emergence of a new absorption band between 1150 and 1190 cm^−1^ as a result of C–O stretching vibrations of the ester group, confirming the cross-linking by an ester bond [23].

The thermal behaviour of the initial PVA and its modified derivatives were investigated with DSC measurements (see Figure S2A in the Supplementary Materials). Due to the conjugation reaction, the melting point values of the conjugated particles PVA-SA-CYS, PVA-SA-MMC, and PVA-SA-CYS-MMC were higher compared to initial PVA [29] because of the formation of crosslinking network that enhances the thermal properties of prepared particles. The water heat evaporation and the desorption enthalpy value were also measured by DSC measurements (see Figure S2B in the Supplementary Materials). The conjugated particles show a lower desorption value at around 29 kJ/mol compared to the initial PVA. The desorption enthalpy of the pure, initial PVA is a little bit higher (43.7 kJ/mol) than the reported vaporization enthalpy of distilled water (41.74 kJ/mol) [33] indicating the hydrophilic nature of the polymer.

### 2.2. Self-Assembled Particle Formation

**Figure 3:**
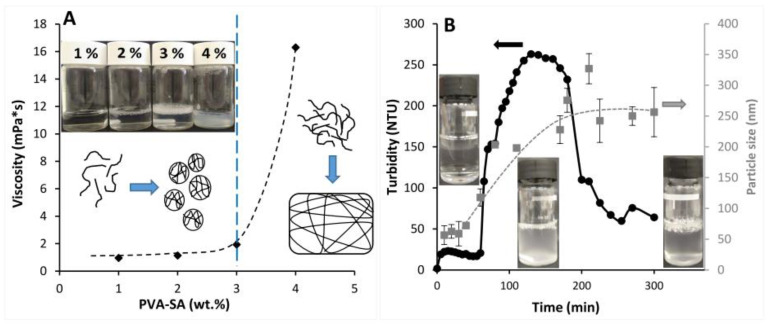
(**A**) The change in the viscosity as a function of increasing the concentration of PVA-SA solutions from increasing the cross-linking ratio. (**B**) The change in the turbidity (black) and particle size (gray) values of PVA-SA with EDC as a function of time from particle formation by ester bond cross-linking.

Before the particle synthesis, we needed to determine the effect of the polymer concentration on the solution properties, since in a dilute aqueous macromolecular solution separate polymeric particles are expected to be obtained from the polymer globule, and the emerging ester cross-links help to maintain their spherical shape. However, if this process takes place in concentrated solutions, a 3D cross-linked structure is expected. Thus, to prepare self-assembled particles, the change in viscosity of PVA-SA solutions was studied to determine the maximum polymer concentration that was suitable to forming separate particles instead of the formation of a cross-linked bulk phase. For this examination, different PVA-SA solutions were prepared followed by adding the proper amount of EDC. Figure 3A shows the change in the viscosity as a function of aqueous polymer concentration. Turbid, low viscosity dispersion was formed in the case of 1, 2, and 3% PVA-SA solutions; however, above this concentration, the viscosity was suddenly increased to 16.3 mPa·s because of the formation of a cross-linked hydrogel. These results indicated that the critical polymer concentration of our modified polymer was around 3% in water; below this concentration separate polymeric particles are obtained, while at higher concentration values the macromolecule chains provide a cross-linked sample.

The self-particle formation was also confirmed by the turbidity measurement, since the simultaneous presence of COOH and OH groups on the polymeric backbone allowed the formation of esters bonds between these functional groups in the presence of EDC. As a result, particles were formed from the homogeneous polymer solution. To study this phenomenon, 2 wt% PVA-SA aqueous solution was prepared and, after adding EDC, the turbidity was monitored as a function of the time, as shown in Figure 3B. A sudden increase in the turbidity was observed after adding EDC (~20 NTU) because activation of the COOH groups of PVA-SA began with EDC, and the turbidity showed a constant value before a significant increase after 60 min of measurement, owing to the self-particle formation by the cross-linking of COOH groups of succinate moieties with OH groups of PVA backbone. After 130 min, the turbidity reached a plateau value and became constant over time until 180 min; after that, it decreased owing to the coagulation of the particles. DLS measurement was performed to check the changes in the size of the prepared particles with time, and the corresponding data are presented in gray in Figure 3B. Particle size showed the tendency to increase with time, and the maximum particle size was ~200 nm during the high turbidity value (between 120 and 170 min), while the maximum particle size was 256 nm after 300 min.

**Figure 4:**
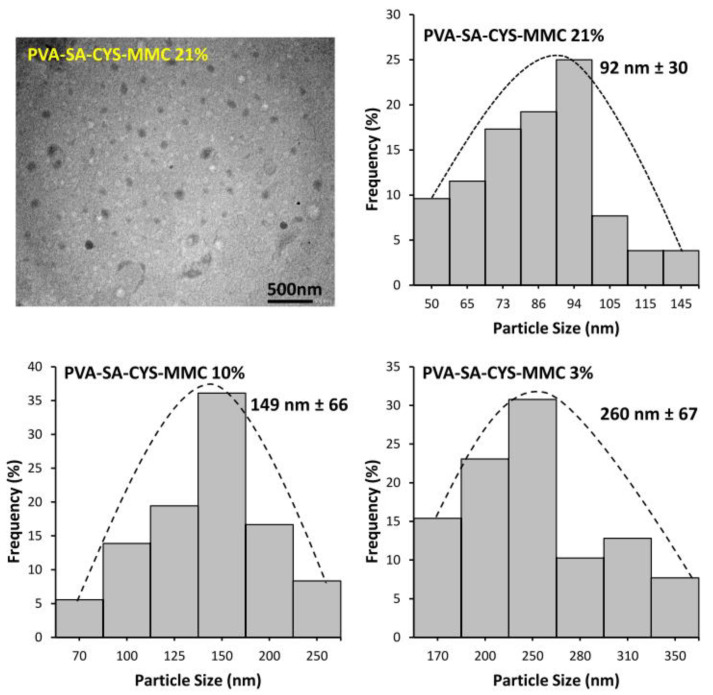
TEM image of prepared polymeric prodrugs (with 21 mol.% cross-linking) with the corresponding size distribution histogram and relative size distribution histograms for PVA-SA-CYS-MMC with 10 and 3 mol.% cross-linking ratios, respectively.

The morphology and the size of the prepared conjugated particles were also studied by TEM. Figure 4 shows a representative TEM image of spherical polymeric prodrug particles (PVA-SA-CYS-MMC 21%) and the corresponding particle size distribution histogram with the mean particle size at around 92 nm. The small particle size of the polymeric prodrug provided an advantage by increasing the surface area and hence increasing the bioavailability of MMC. The enhanced particle surface area is also very important for the mucoadhesive properties of a polymeric prodrug by helping to increase the adhesion of particles to the mucous membrane, as we will see in the mucoadhesive measurement section. Figure 4 also shows the corresponding particle size distribution histograms for PVA-SA-CYS-MMC (10%) and PVA-SA-CYS-MMC (3%) with mean particle sizes at around 149 nm ± 66 and 260 nm ± 67, respectively. It can be seen that the increasing cross-linking density resulted in a decrease in the particle size of the prepared polymeric prodrugs.

### 2.3. Mucoadhesive Properties of Cysteamine Polymeric Conjugate

The required mucoadhesive properties of the formed particles were ensured by the terminal thiol groups. The thiol content values determined by Volhard’s method (see the Supplementary Materials) were 0.8, 0.2, and 0.075 mmol/g in the case of PVA-SA-CYS samples with ~21, 10, and 3% cross-linking densities, respectively.

These terminal thiol groups enabled the particles to form covalent S-S bonds with the SH groups of mucin molecules [15,16]. This mechanism was verified via rheological measurements since the mixing of 1% *w*/*v* PVA-SA-CYS solution with 1% *w*/*v* mucin solution resulted in a viscous cross-linking hydrogel (Figure S4). The storage modulus (G′) provides information about the elastic nature of the polymer while the loss modulus (G″) represents the viscous property. Figure S5 shows that the G′ of PVA-CYS-mucin varied from 10^4^ to 10^5^ Pa but the G″ of PVA-CYS-mucin varied from 10^3^ to 10^4^ Pa. The ‘sum’ of the loss and storage modulus is the so-called complex modulus (G*). According to Table 1, the PVA-CYS-mucin suspension had much higher G′ values (158.3 kPa) than PVA-CYS (24.7 kPa) and mucin (12.4 kPa), mainly because of the formation of a more entangled network by hydrogen bonds and disulfide linkages, wherein the thiolate polymer served as a cross-linker that led to the formation of the gel (as shown in Figure S4), which was consistent with the literature [34]. With the same approach, the loss modulus of PVA-CYS-mucin had higher values (23.2 kPa) than PVA-CYS (7.55 kPa) and mucin (0.78 kPa).

**Figure 5:**
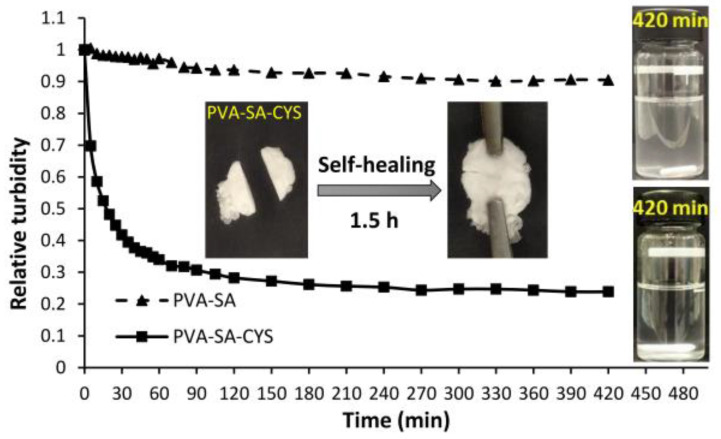
The change in the relative turbidity of the 1 wt% aqueous particle dispersion due to the particle adsorption on the surface of the 7 cm pig intestinal membrane immersed in the dispersion. The inserted photos on the right show the aqueous particle dispersions after the end of the measurement and the inserted photos in the middle represent the self-healing test for PVA-SA-CYS that was cut into two parts and reattached.

The formation of the disulfide bonds between our mucoadhesive polymer particles and the mucin-rich surface was also demonstrated by surface adsorption measurements. During this experiment, a pig intestinal membrane as the model mucus surface was immersed in 1 wt% of a gently agitated aqueous dispersion of particles, and the decrease in the turbidity due to the particles’ adsorption was measured as a function of time. The same measurement was repeated with particles without CYS as control, as shown in Figure 5. The presence of CYS increased the mucoadhesive properties of the initial PVA. The turbidity decreased dramatically over time because of the continuous surface adsorption of the mucoadhesive particles (PVA-SA-CYS 21%) on the intestinal membrane until nearly full adsorption was reached, with a maximum decrease in the turbidity value of approximately 76.2% compared to the initial PVA-SA particles with maximum decrease of 9.5% after 7 h. After 24 h, the drop in turbidity value in the PVA-SA-CYS example remained essentially steady, confirming the permanence of the surface bonding state. In the case of PVA-SA-CYS, around 60% of the particles were adsorbed on the intestinal surface after 1 h of measurement, resulting in the increasing residence time of the conjugated particles and improved bioavailability [35]. It is worth noting that because of the presence of pendant SH groups, the PVA-SA-CYS macromolecules can also form disulfide bonds on their own. The inserted photos in Figure 5 show the intelligent self-healing property of this polymer, where the test sample (PVA-SA-CYS 21%) was cut in half and reattached. The evolving disulfide bonds resulted in a recovered, cross-linked structure from the two parts.

### 2.4. In Vitro Drug Release Measurement

**Figure 6:**
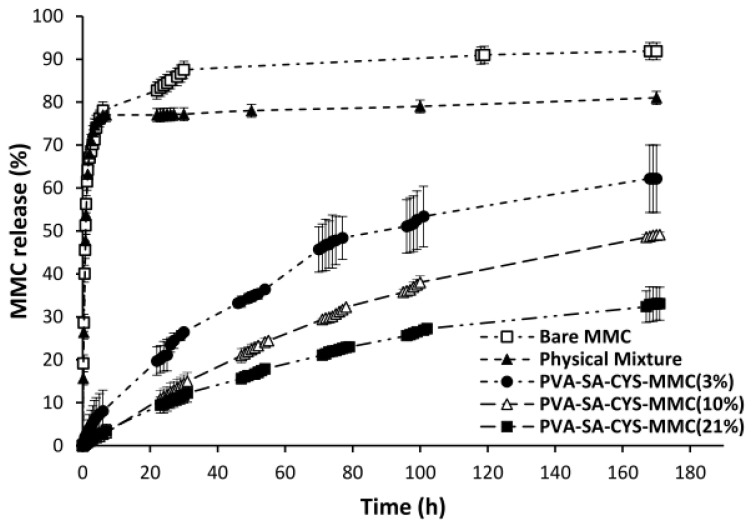
The in vitro releasing kinetic curves of pure MMC, MMC from the physical mixture, and PVA-SA-CYS-MMC with different cross-linking densities.

The profiles of MMC released from different forms are shown in Figure 6; release from the bare MMC and the physical mixture was too fast a process with maximum releasing amounts of 92 and 81%, respectively.

According to Table S1, releases from the bare MMC and the physical mixture of MMC with polymer data were fitted with the Korsmeyer and Peppas model. Korsmeyer and Peppas developed a basic relationship that explains the release of drugs from a polymer system that follows the form of dissolution and is defined as Equation (1) [36]:

M_t_/M_∞_ = Kt^n^ (1)

where M_t_/M_∞_ denotes the percentage of drug released at time t, n denotes the release exponent (or diffusional exponent) and k is the release rate constant. Based on an “n” value for spherical geometry, the release exponent n ≤ 0.43 indicated a Fickian diffusion mechanism, whereas 0.43 < n < 0.85 corresponded to non-Fickian (anomalous) transport [36,37]. Because n < 0.2 for the bare MMC and the physical mixture of MMC, this implied a Fickian diffusion release, i.e., diffusion-controlled transport.

In contrast, the release of MMC from the conjugated form (PVA-SA-CYS-MMC) with different cross-linking densities (3, 10, and 21%, respectively) showed prolonged-releasing profiles determined by the amide hydrolysis of the MMC moiety from the conjugated form. Amide hydrolysis reactions were very slow and therefore took a long time to reach the plateau value; the maximum MMC releasing values after 7 days for PVA-SA-CYS-MMC (21%), PVA-SA-CYS-MMC (10%) and PVA-SA-CYS-MMC (3%) were 33.1, 49.1 and 62.2%, respectively. According to Table S1, drug release data for all the conjugated forms were fitted with the Korsmeyer–Peppas model. Based on an “n” value (diffusional exponent) ranging between 0.43 and 0.85, which correlates to an anomalous transport (non-Fickian), the drug release mechanism was dependent on both diffusion and erosion-controlled mechanisms [38,39,40,41].

### 2.5. In Vitro Evaluation of the Anticancer and Antibacterial Activity of MMC Samples

The anticancer activity of the MMC and its conjugated forms was also studied. After 1, 3, 5, and 7 days, the released medium with the given MMC concentration (see Table 2) was withdrawn and the IC_50_ values were determined (Table 2). All the MMC-containing samples showed an obvious antitumor effect; the measured IC_50_ values varied between 0.014 ± 0.001 and 0.054 ± 0.029 mg/mL. The IC_50_ values of the encapsulated samples were very similar to the values of pure MMC in each case, indicating that neither the conjugation of the MMC nor the dissolution of the drug from the particles caused an adverse change in the effect of the drug. These results indicated that the polymeric particles were able to efficiently adhere to mucus surfaces, and because of the prolonged release, the anticancer effect of MMC could be felt for a long time. Moreover, the developed system had antibacterial properties.

**Figure 7:**
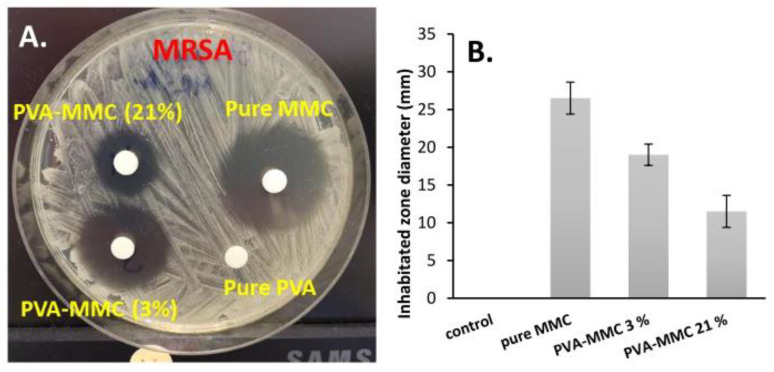
Antibacterial effects of pure MMC and encapsulated forms on Methicillin-resistant *Staphylococcus aureus (MRSA)* test bacteria (**A**), with the obtained diameter values of the corresponding specific inhibition zone (**B**). Control: polymeric particle without MMC.

Figure 7 shows a representative result obtained after 5 days of dissolution of MMC against MRSA. We noted that both pure MMC and encapsulated forms inhibited the growth of MRSA bacteria at the measured doses because the diameter of the inhibition zones was already more than 10 mm. Figure S6 shows the obtained diameter values of the corresponding specific inhibition zone after 7 days of antibacterial effects. Furthermore, the antibacterial effect (size of inhibition zones) was well correlated with the released amount/concentration of MMC because the highest effect was obtained in the case of pure MMC and the diameters were decreased with the increasing cross-link densities.

## 3. Discussion

In this work, polymeric prodrug nanoparticles with spontaneous, self-assembled particle formation ability and mucoadhesive properties were used to reduce the severe side effect of MMC. The particles were synthesized by conjugation of MMC and CYS with succinated PVA combined with self-assembled particle formation using EDC in a simple one-pot reaction, as shown in Figure 1.

The successful and varied (3–21%) substitution degree of PVA and coupling reactions were confirmed using FTIR (Figure 2) and EDX (Figure S3) measurements, while the self-assembled particle formation process was proved by turbidimetric measurements (Figure 3B). The size and morphology of polymeric prodrug nanoparticles were examined by TEM measurements that showed spherical nanoparticles with the mean particle size ranging between ~92 to 260 nm, depending on the cross-linking density (3–21%) (Figure 4).

The mucoadhesive properties of the conjugated particles showed strong attachment with the pig intestinal test membrane that was demonstrated by turbidity measurement and high values of storage modulus (G′ = 158.3 kPa) and loss modulus (G″ = 23.2 kPa) when mixing with porcine mucin. This led to the formation of a cross-linking hydrogel by a disulfide bond between a thiol group of polymer and cysteine-rich sub-domains of the mucus glycoproteins (mucin) (Figure S4) [15,16]. The prepared conjugated particles with strong mucoadhesive properties are suitable for increasing the residence time of the polymeric prodrug of MMC within the human body during the prolonged process of MMC release and improved bioavailability [35].

The in vitro drug release experiments were performed for 7 days under physiological conditions (PBS, pH = 7.4) that indicated the drug-releasing process can be adjusted by variation of the cross-linking density. PVA-SA-CYS-MMC (21%) was the slowest one with maximum drug release of 33.1% compared to 62.2% for PVA-SA-CYS-MMC (3%) (Figure 6), and all MMC release data for polymeric prodrugs were fitted with the Korsmeyer–Peppas model (Table S1) [36] where the drug-releasing mechanism depended on both diffusion- and erosion-controlled mechanisms [38,39,40,41]. This means the hydrolysis reaction of the conjugated form first, followed by diffusion of the Mitomycin drug. Thus, it can be concluded that the release rate of MMC can be significantly prolonged by the application of our modified polymer; furthermore, the amount and kinetics of the released drug can be adjusted by the synthesis condition.

Because of the control of MMC release by cross-linking density, the antibacterial effect (the diameter of the inhibition zones) was dependent on the released amount/concentration of the MMC where the diameters were decreased (26.5, 19, and 11.5 mm) with the increasing cross-link densities (0/pure MMC/, 3 and 21%) (Figure 7). The anticancer effect of the polymeric prodrugs was very similar to the values of pure MMC in each case, which means the effect of MMC was not affected by conjugation with the polymer and dissolution from the particle system (Table 2). Eventually, we hope that the prepared polymeric prodrug particles of MMC will become a promising drug delivery system (DDS) and useful in the area of throat cancer treatment.

## 4. Materials and Methods

The Supplementary Materials contain a description of the materials as well as some of the synthetic procedures (e.g., preparation of the succinic anhydride precursor according to [42], preparation of PVA-SA according to [43] with modification of reported procedures) and characterization methods (e.g., thiol content determination by Volhard’s silver nitrate method [44], mucoadhesive measurement, anticancer activity, and antibacterial activity using the standard disk diffusion technique [45], etc.).

### 4.1. Preparation of PVA-SA-CYS-MMC by One-Pot Reaction

The polymeric prodrug of MMC as shown in Figure 1 was prepared according to the following recipe: 100 mg of PVA-SA with different carboxylic contents (0.5, 1.65, and 3.4 mmol/g of the COOH group; 3.1, 10.4, and 21.5% substitution degrees, respectively, as shown in Figure S1B) was dissolved in 10 mL of distilled water, then 1.5 equivalent of EDC to COOH content (14.4, 47.4, and 97.8 mg; 0.075, 0.248, and 0.51 mmol, respectively), 4 mg of MMC, and 1 equivalent of cysteamine to COOH content (3.9, 7.3, and 9.6 mg; 0.05, 0.165, and 0.34 mmol, respectively) was added to the polymer solution and left for 3 h under magnetic stirring at room temperature. After the conjugation and self-assembled particle formation, the reaction mixture was added to an excess amount of ethanol to precipitate the conjugated polymeric particles product followed by centrifugation to remove the solvent and washing with a mixture of acetone and water in a 3:1 ratio. The precipitate was left to dry under the vacuum, the absorbance of the supernatant was measured by UV-spectroscopy to determine unbound MMC and the percent of MMC binding was calculated according to Equation (2).

(2) $$\mathrm{Percent}\;\mathrm{of}\;\mathrm{binding}\;\left(\%\right)=\frac{\mathrm{Total}\;\mathrm{MMC}-\mathrm{unbound}\;\mathrm{MMC}}{\mathrm{Total}\;\mathrm{MMC}}\times 100.$$

### 4.2. Methods of Characterization

The FTIR spectroscopy measurements were carried out between 4000 and 500 cm^−1^ through the accumulation of 128 scans at 1 cm^−1^ resolution utilizing the BioRad FTS-60A FTIR spectrometer (Bio-Rad, Krefeld, Germany). Thermo Scientific GRAMS/AI Suite software was used for all spectral manipulations.

A transmission electron microscope (TEM) was used to investigate the morphology and particle size of the prepared conjugated particles. The investigation was carried out utilizing a Philips CM-10 transmission electron microscope with a 100 kV acceleration voltage. For TEM measurements, the dispersed samples were disseminated and dried on lacey carbon films on copper grids with 200 mesh.

Viscosity measurements were carried out using a Physica MCR 301 rheometer (Anton Paar, Graz, Austria) equipped with a roller geometry cylinder type (DG 26.7) and the measurements were performed at 25 °C. Aqueous PVA-SA (21.5% cross-linking density) solutions were prepared at different concentrations (1, 2, 3, and 4 wt%), then 1.5 equivalent of EDC to COOH content was added to the prepared solutions, and the change in the viscosity was measured 15 min after addition of EDC.

The self-assembled particle formation of liner macromolecules through ester bonds was studied based on the turbidity measurement that was performed using an ISO Portable Turbidity Meter—HI98703 (Hanna instruments, Cluj-Napoca, Romania). A 2 wt% amount of PVA-SA (with 21.5% cross-linking density) aqueous solution was prepared and 1.5 molar equivalent of EDC to COOH content was added, followed by monitoring the change in the turbidity over time and simultaneously measuring the particle size by DLS (SZ-100 HORIBA Scientific, HORIBA, Ltd., Kyoto, Japan).

The in vitro experiments on MMC release were performed using a cellulose membrane (Sigma-Aldrich (St. Louis, MO, USA), avg. flat width 10 mm (0.4 in.)). The MMC amounts released from the bare and conjugated forms were spectrophotometrically measured by monitoring the change in the intensity of the characteristic absorbance peak of MMC at λ = 364 nm. The MMC concentration was directly proportional to the maximal absorption values measured at this wavelength [C_MMC_ (mg/mL) = (A_364 nm_)/20.418; R^2^ = 0.9998], according to the calibration curve previously determined within the range of 0–0.08 mg/mL MMC solution. Solid powders of the pure drug and conjugated forms (equivalent to 0.057 mg/mL MMC) were inserted in a cellulose membrane that was closed and placed into 20 mL of a PBS buffer solution at pH = 7.4 (37 °C; 0.9 wt% NaCl content). In order to determine the effect of the conjugation of MMC to polymer in the releasing process, the release of MMC from a physical mixture of pure MMC and PVA-SA particles (with the same ratio as the conjugated particles) was studied. During the measurement, 3 mL of the aqueous solution was taken from the release medium at selected time intervals and the average MMC concentration with the corresponding standard deviations was determined.

## Figures and Tables

**Table 1:** Data of storage modulus (G′), loss modulus (G″), and complex modulus (G*) of PVA-SA-CYS (1%), mucin (1%), and a mixture of them.

Sample	G′ (kPa)	G″ (kPa)	G* (kPa)
1% PVA-CYS	24.7	7.55	32.25
1% Mucin	12.4	0.78	13.18
1% PVA-CYS-Mucin	158.3	23.2	181.5

**Table 2:** The spectrophotometrically measured MMC concentration values (in mg/mL) of the solutions used during the antitumor and antibacterial tests, the determined IC_50_ values (in mg/mL) of the tested samples released from the encapsulated and pure MMC on the Hep-2c tumor cell line.

Sample	1st Day		3rd Day		5th Day		7th Day	
	Conc (mg/mL)	IC_50_ (mg/mL)	Conc (mg/mL)	IC_50_ (mg/mL)	Conc (mg/mL)	IC_50_ (mg/mL)	Conc (mg/mL)	IC_50_ (mg/mL)
Pure MMC	0.254	0.029 ± 0.001	0.270	0.041 ± 0.002	0.273	0.026 ± 0.006	0.275	0.05 ± 0.045
Control	0.000	0.000	0.000	0.000	0.000	0.000	0.000	0.000
PVA-MMC (3%)	0.063	0.014 ± 0.001	0.140	0.022 ± 0.007	0.173	0.043 ± 0.025	0.188	0.054 ± 0.029
PVA-MMC (21%)	0.029	0.037 ± 0.004	0.065	0.038 ± 0.01	0.089	0.039 ± 0.007	0.097	0.029 ± 0.004

## Data Availability

Not applicable.

## Supplementary Materials

The following supporting information can be downloaded at: www.mdpi.com/article/10.3390/ijms23126807/s1.
