# Supplementary material for: Use of Self-Assembled Colloidal Prodrug Nanoparticles for Controlled Drug Delivery of Anticancer, Antifibrotic and Antibacterial Mitomycin
Source: Int J Mol Sci. 2022 Jun 18;23(12):6807. doi: 10.3390/ijms23126807 (PMC9224153; doi:10.3390/ijms23126807)
Supplement: Supplementary file 1 [file ijms-23-06807-s001.zip › ijms-1774420-supplementary.pdf]

International Journal of *Molecular Sciences*

MDPI

Supplementary information

Use of self-assembled colloidal prodrug nanoparticles for controlled drug delivery of anticancer, antifibrotic and antibacterial mitomycin

Mohamed M. Abdelghafour[1,2], Ágota Deák[1], Diána Szabó[3], Imre Dékány[1], László Rovó[3], László Janovák[1,*]

[1] Department of Physical Chemistry and Materials Science, University of Szeged, Rerrich Béla tér 1, H-6720 Szeged, Hungary; m.abdelghafour2015@yahoo.com (M.M.A.); dagota13@yahoo.com (Á.D.); i.dekany@chem.u-szeged.hu (I.D.)
[2] Department of Chemistry, Faculty of Science, Zagazig University, Zagazig 44519, Egypt
[3] Department of Oto-Rhino-Laryngology and Head & Neck Surgery, University of Szeged, Tisza Lajos krt. 111, H-6724 Szeged, Hungary; diniklinik@freemail.hu (D.S.); office.orl@med.u-szeged.hu (L.R.)
* Correspondence: janovakl@chem.u-szeged.hu; Tel.: +36-62-544-210; Fax: +36-62-544-042

Materials

Polyvinyl alcohol (PVA, 86-89% hydrolyzed) was purchased from Nagart Kft., Hungary. Mitomycin C was purchased from 1Pluschem LLC., San Diego, CA 92121, USA. Succinic acid ($C_4H_6O_4$), acetic anhydride ($C_4H_6O_3$), sodium acetate ($C_2H_3NaO_2$), sodium hydroxide (NaOH), and acetone (C_3H_6O) were acquired from Molar Chemicals Kft., Hungary, while 1-(3-Dimethylaminopropyl)-3-ethylcarbodiimide hydrochloride (EDC, $C_8H_{17}N_3 \cdot HCl$, Mw 191.70, 98+%) from Alfa Aesar. Phosphate-buffered saline (PBS; pH ~ 7.4) solution was prepared using sodium dihydrogen phosphate monohydrate ($NaH_2PO_4 \cdot H_2O$) from Sigma-Aldrich, as well as di-sodium hydrogen phosphate dodecahydrate ($Na_2HPO_4 \cdot 12H_2O$) and sodium chloride (NaCl) acquired from Molar Chemicals Kft., Hungary. DMF and diethyl ether were obtained from Fluka and VWR Chemicals BDH®, respectively. Mucin from the porcine stomach (type III) for mucoadhesive measurements was purchased from Sigma Aldrich. All chemicals were used as received without further purification.

Chemistry

Preparation of succinic anhydride precursor

The succinic anhydride was synthesized according to the following procedures [43], briefly, 15 g of succinic acid was mixed well with 25 ml of acetic anhydride under nitrogen atmosphere in a 100 ml round-bottom flask equipped with an anhydrous calcium chloride tube closed reflux condenser. The reaction mixture was gently heated with occasional shaking on the steam bath until a clear solution was obtained and then left for 1 h to ensure the reaction was complete. The flask was cooled to room temperature and then placed in an ice bath, the obtained white crystals were collected by filtration, then washed with a small amount of diethyl ether, and then dried under vacuum. The collected product was 10.3 g with the percent of yield equal to 81.1%.

Preparation of PVA-SA

The functionalization of PVA with succinic anhydride (Figure 1) was carried out according to [44] with the modification of the reported procedures. 1 g of PVA with the different molar ratios (8, 16, 24, 32, 63.3, 95, and 126.6 molar% which are equivalent to 0.126, 0.25, 0.38, 0.5, 1, 1.5, 2 g, respectively) of succinic anhydride for OH content of PVA

was added to 8.6 ml of DMF followed by the addition of 0.05 g of anhydrous sodium acetate as catalyst. The reaction mixture was left under continuous magnetic stirring at 45 °C for a duration of up to 24 h. Then, an excess of diethyl ether was used to precipitate the reaction product. The obtained crude product was subsequently purified by repeated dissolving in DMF and precipitation in an excess amount of ether several times then washed with ether and then dried under vacuum until constant weight.

Characterization methods

To determine the available carboxyl group in the succinate polymer (PVA-SA), acid-base titration was performed by dissolving 0.1 g of PVA-SA in 10 ml of distilled water under magnetic stirring and warming to obtain a fully clear solution. The prepared solution was titrated against 0.1 M of NaOH in the presence of a phenolphthalein indicator at room temperature. To evaluate the standard deviation, the titration procedure was repeated three times (presented as error bars).

Molecular weight determination of initial PVA was performed by using dynamic light scattering (DLS) measurements. The measurements were performed at a fixed scattering angle (90°) with different polymer concentrations (1-10 mg/mL) via using a Horiba SZ-100 Nanoparticle analyzer. All the prepared solutions were filtered using 0.22 µm Millipore membrane filters before the measurements. Toluene was used as a standard to achieve the absolute scattering light intensity. According to the Rayleigh equation (Equation (S1)) and Debye plot formulation, the Mw can be calculated by the angle (θ) and concentration (C) dependence of $KC/\Delta R_\theta$.

$$\frac{KC}{R_\theta} = \left(\frac{1}{M} + 2A_2C\right) \tag{S1}$$

where K denotes an optical constant, C means the sample concentration, R_θ indicates the Rayleigh ratio of the scattered to incident light intensity, M denotes the weight-average molecular weight, and A_2 indicates the second virial coefficient.

Differential scanning calorimetry (DSC) measurements using a Mettler-Toledo 822e instrument were used to evaluate the thermal behaviour of the initial PVA and its modified derivatives. The measurements were performed on dried polymer samples that were heated from 25 to 500 °C at a rate of 5 °C/min. Besides these measurements, the water desorption enthalpy values for the swollen hydrogels were also measured by DSC. The samples were swelled in distilled water to prepare 2 wt.% hydrogels and left overnight to achieve the swelling/dissolution equilibrium. Using an Eppendorf miniSpin instrument, the prepared solutions were centrifuged at 14500 rpm for 10 min, the supernatant was removed, and the sediment (concentrated polymer gel) was measured. These measurements were performed in the temperature range of 25–200 °C with a 5 °C/min heating rate. DSC measurements were repeated three times; the standard deviation is presented as error bars.

Energy-dispersive X-ray (EDX) measurements were performed using the Röntec EDS detector at 20 keV for elemental composition analysis of PVA-SA and conjugated forms, the morphology was examined by field emission scanning electron microscopy (SEM–Hitachi S-4700 microscope) with 20 kV acceleration voltage. Dried powder samples were used for this measurement and placed in the sample holder.

Water contact angle (Θ) measurements were evaluated by using of drop shape analysis (Easy Drop, Krüss GmbH) system equipped with a 0.5 mm needle syringe to introduce the water drops on the surface of prepared polymeric films at 25.0±0.5 °C. Utilizing the CCD camera goniometer, the Young – Laplace equation was used to mathematically characterize the drop contour of the captured photo using DSA100 software via using DSA100 software, and the Θ was described as the slope of the contour line at the point of contact of three phases. Thin films of samples were prepared on glass sheets using a spray drying technique using water as a medium.

Thiol content determination by Volhard's silver nitrate method

100 mg of cysteamine thiolated PVA with mucoadhesive properties (PVA-SA-CYS) was added to 25 ml of 0.01 M silver nitrate solution. The reaction mixture was covered well to avoid the light and stirred for 4 h before titration. After that time, unreacted silver nitrate was determined by titration with standardized 0.01 M KSCN using ferric nitrate as an indicator. The endpoint of the titration was estimated when forming of the red colour complex $[Fe(SCN)_6^{3-}]$ from an excess of thiocyanate anion (SCN^-) and the ferric ion (Fe^{3+}) of the indicator [45]. The amount of polymer's thiol content was calculated from the amount of unreacted silver ion according to (Equation (S2)).

$$\text{Thiol content (mmol/g)} = \frac{0.25 - (C * V)}{\text{wt of PVA}} \quad \text{(S2)}$$

Mucoadhesive measurement

Mucoadhesive properties of PVA-SA-CYS were evaluated via oscillatory rheology measurements using a Physica MCR 301 rheometer (Anton Paar, Graz, Austria) featuring cone/plate type (cone angle 0.99°, gap height in the middle of the cone 0.054 mm, and diameter 11.95 mm) system. During the measurement, 1% w/v of PVA-SA-CYS and 1% w/v of mucin aqueous solutions were prepared, the solutions were mixed in the same proportions, and the gelation was measured after overnight. The gelation of the PVA-SA-CYS, mucin, and mixture was followed at an angular frequency of 10 Hz at 25 °C, and storage modulus (G') and loss modulus (G") were evaluated over the strain range from 0.1 to 10%.

The mucoadhesive properties of the polymeric particles were also evidenced via a simple adsorption experiment. 10 ml of 1 wt.% aqueous solution was prepared from the polymeric particles (PVA-SA and PVA-SA-CYS) and a 7 cm long pig intestine obtained from a local slaughterhouse was immersed in these turbid dispersions. The prepared dispersion was stirred with a speed of 200 rpm and the decrease in the turbidity caused by the surface adsorption of particles on the intestine membrane was measured as a function of time with an ISO Portable Turbidity Meter - HI98703 (Hanna instruments).

Anticancer and antibacterial activity

The anticancer properties of the MMC and encapsulated MMC was also measured and presented. During these test experiments, 3 mg MMC (free or encapsulated form) was put in the cellulose membrane (Sigma-Aldrich, avg. flat width 10 mm (0.4 in.)) and it was immersed in 10 ml of PBS (pH 7.4, 37°C) buffer solution and the samples were gently agitated. After 1, 3, 5, and 7 days, 0.5 mL of the releasing medium was taken and the IC50 values were determined by cytotoxicity test in 96-well cell culture microplates with 3-(4,5-dimethylthiazol-2-yl)-2,5-diphenyltetrazolium bromide (MTT) assay. Hep-2c human carcinoma cells were seeded at a density of 1×104 cells/well and incubated for 24 h at 37°C. After 24 h, the tested MMC and encapsulated forms were serially half diluted in EMEM medium in microtiter plates. In 100 µl of the medium, the compounds were diluted. In parallel, the concentration of MMC was also measured spectrophotometrically. After dilution, in medium diluted compounds were added to Hep-2c cells to each well, except for the medium control wells. After a 24 h incubation period at 37°C, each well was injected with 20 µl of (5 mg/ml) MTT solution (Sigma-Aldrich, Germany). Following a 4 h incubation at 37°C, 100 µl of 10% sodium dodecyl sulfate (SDS, Sigma-Aldrich) was poured into each well for dissolving the formed formazan, then the optical density (OD) was recorded after 24 h. The cell growth was estimated by determining the OD at 540 nm (ref. 630 nm) using a Multiscan EX ELISA reader (Thermo Lab systems, Cheshire, WA, USA). In the experiment, the solvent had no influence on cell growth at the doses utilized for IC_{50}

calculations. GraphPad Prism software (GraphPad Software, San Diego, USA) version 5.00 for Windows with nonlinear regression curve fitting was used to calculate the IC_{50} values and the standard deviations of triplicate experiments.

The in vitro evaluation of the antibacterial activity of the MMC solution obtained from the above-described drug release experiments was also determined. The antibacterial activity of the MMC against the Methicillin-resistant Staphylococcus aureus (MRSA) bacterial strains was evaluated via estimating their inhibition zones using the standard disk diffusion technique [46]. The sterile filter paper (D = 6 mm) discs soaked with the solutions (10 µl of solutions) were put on Tryptic Soy Agar plates (Sigma) and inoculated with the relevant bacterial suspensions (inocula of 0.5 McFarland's standard). As a bacterial growth control, another plate was inoculated without disks. Within 15 min of inoculating the plates, the disks were placed, and incubation began within 15 min of the disks being applied (in accordance with EUCAST standards) [46]. For 24 h at 37°C, the plates were incubated. The diameters of the inhibition zones (Minimum Inhibitory Concentrations – MICs) resulting from the studied compounds (including disc diameter) were measured. Once the width of the inhibition zones in the measured concentrations was less than 10 mm, a chemical was deemed inactive. All experiments were carried out in triplicate.

Results and discussion

According to the DLS measurements and Debey plot determination, the molecular weight (M_w) of the initial PVA was 46.83 kDa (Figure S1A). Figure S1B shows the substitution degree as a function of increasing the molar ratio of SA. The percentage of substitution increases with increasing molar ratio of SA until reaches the maximum (saturation) value at around 21.5% substitution at 90 molar% of applied SA. Due to the mild reaction condition and the applied DMF as a poor solvent for PVA, the percent of substitution was relatively low because the reaction only occurred on the surface of partially dissolved PVA particles, however, using extreme conditions for substitution reaction will lead to the crosslinking of PVA by SA which was not preferred for our work as was studied by Zhou et al. [26].

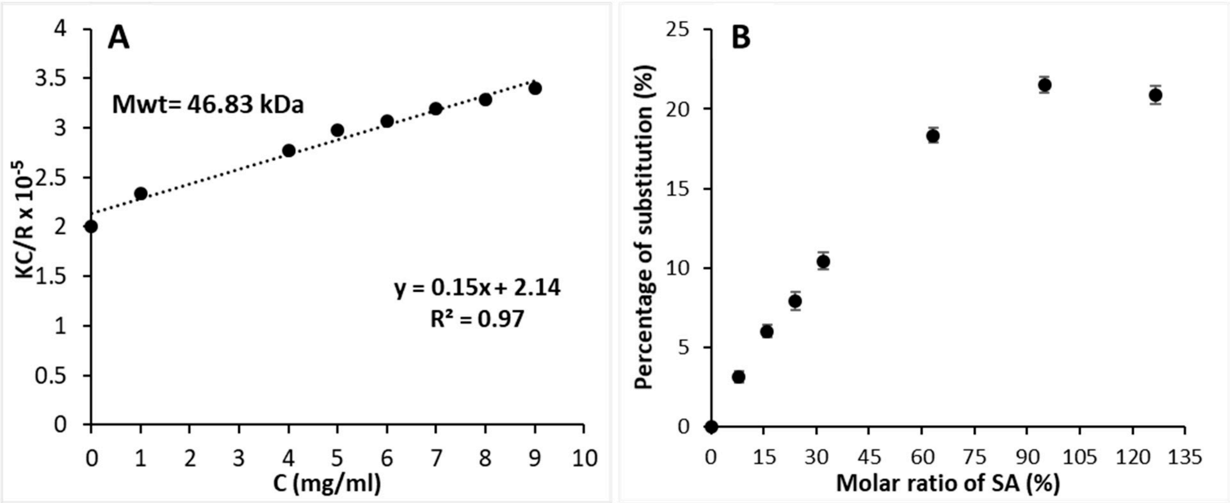

Figure S1. Debye plot to measure the molecular weight of initial PVA (**A**), the substitution degree of PVA-SA samples as a function of molar ratio (%) of reacted succinic anhydride (SA) with OH of PVA (**B**)

The thermal behaviour of the initial PVA and its modified derivatives were investigated with DSC measurements. Figure S2A shows the DSC curves of initial polymer and conjugated particles. The initial PVA has three different endothermic peaks at ~80, 190, and 310 °C, which corresponds to the water loss, melting, and thermal decomposition of the polymer, respectively [33]. The melting point of conjugated particles is higher (235, 240, and 230 °C for PVA-SA-CYS, PVA-SA-MMC, and PVA-SA-CYS-MMC, respectively) compared to the initial PVA (190 °C) due to the formation of a crosslinking network that enhances the thermal properties of prepared particles in addition to increase the hydrophobicity that was also confirmed by water contact angle measurement on the prepared films as shown in the inserted photo in Figure S2B. The conjugated particles (PVA-SA-CYS) show a high contact angle at around 91° but in the case of initial PVA and succinate PVA samples, only 55° and 57° were measured, respectively. This is presumably due to the consumption of the hydrophilic (OH and COOH) groups of the initial polymer.

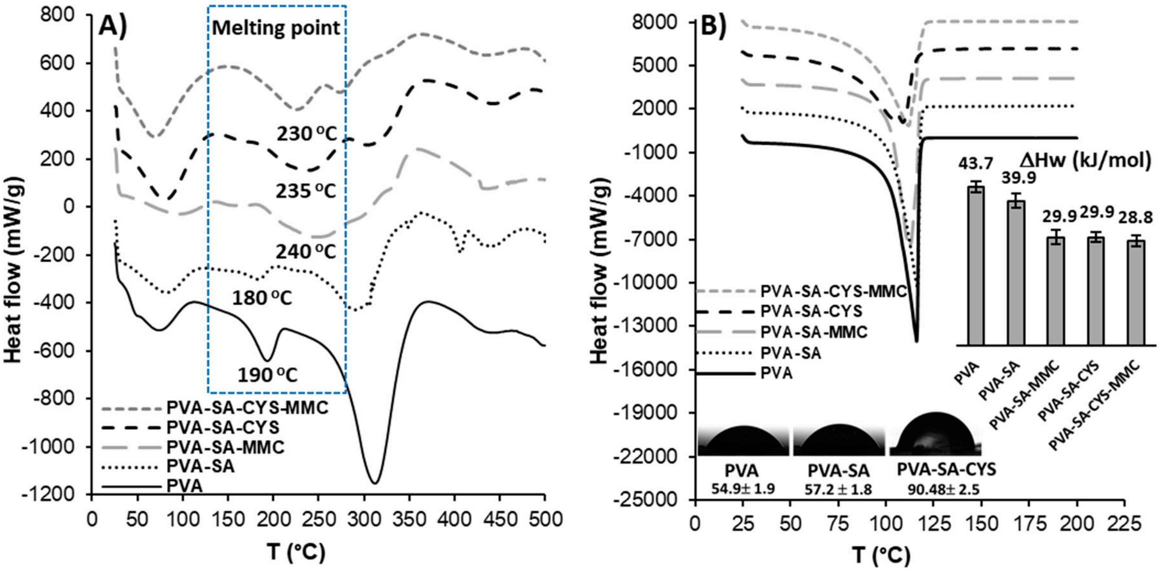

Figure S2. A) DSC curve of initial PVA, PVA-SA, and conjugated particles, **B**) curves of the PVA-based hydrogel samples with the determined water desorption enthalpy (ΔH_w) values (grey columns). The contact angle measurement of films was represented as the inserted picture.

To prove the change in the hydrophobization besides the contact angle measurement and further recognize the changes in the thermal properties, the water heat evaporation and the desorption enthalpy value were also measured by DSC measurements. Figure S2B shows the endothermic peaks corresponding to the water evaporation obtained during the heating of the swollen samples and the determined vaporization enthalpy values (ΔH_w, grey columns). The desorption enthalpy of the pure, initial PVA is a little bit higher (43.7 kJ/mol) than the reported vaporization enthalpy of distilled water (41.74 kJ/mol) [34] indicating the hydrophilic nature of the polymer. The conjugated particles show a lower desorption value at around 29 kJ/mol compared to the initial PVA and succinate PVA with values at around 43.7 and 39.9 kJ/mol, respectively. To conclude the obtained results from thermal measurements, the coupling reaction of anticancer agent and aminothiol in addition to crosslinking slightly increased the hydrophobicity of prepared polymeric prodrug that led to enhanced thermal properties. This increased hydrophobicity is advantageous from the viewpoint of particle formation because the macromolecule chains with lower water solubility result in smaller polymer globules in the aqueous environment and thus the formed polymeric particles are also smaller.

EDX measurements were also used to detect the presence of the elements in the different conjugated forms, PVA-SA-CYS particles show the appearance of the sulfur and nitrogen element that confirms the conjugation of CYS to PVA-SA by coupling reaction with EDC. In the case of PVA-SA-CYS-MMC, the sulfur and nitrogen elements also appeared due to CYS and MMC that contain nitrogen in addition to carbon as shown in Figure S3.

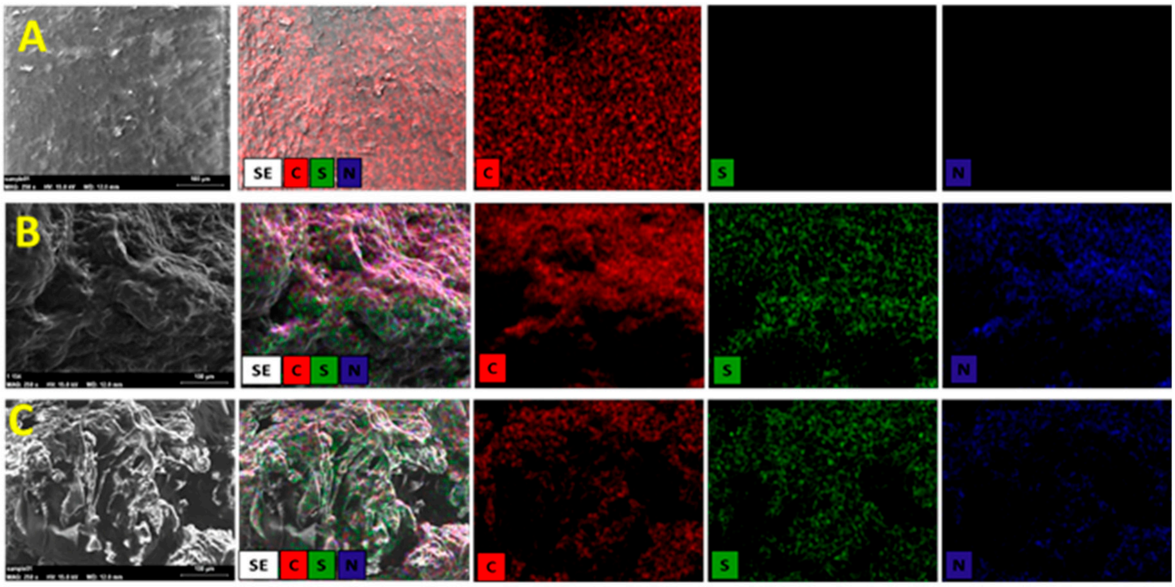

Figure S3. EDX of (**A**) cross-linking PVA-SA and (**B**) PVA-SA- CYS as well as **C)** PVA-SA-CYS-MMC (Scale bar 100μm)

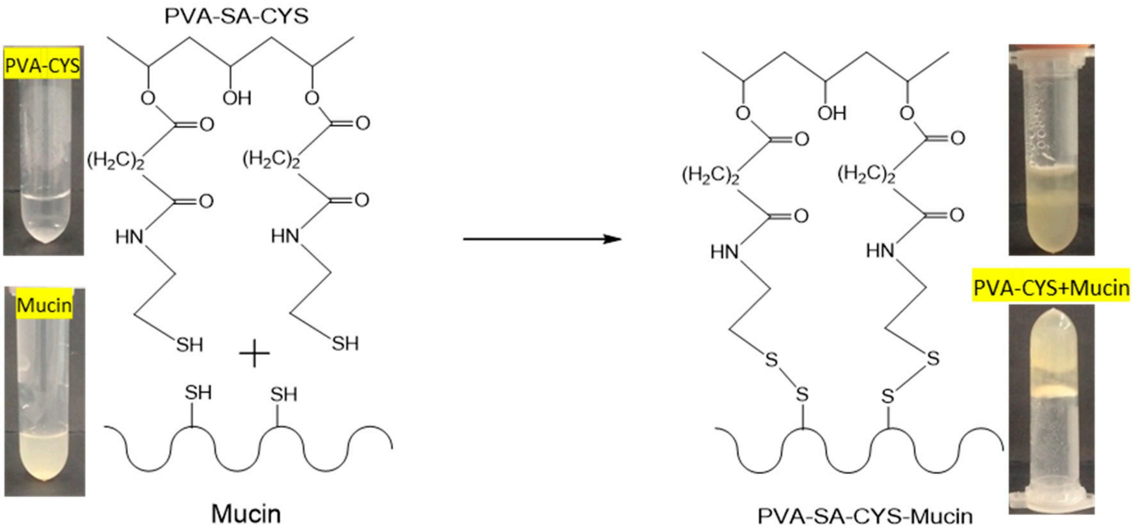

Figure S4. Scheme of the crosslinking of PVA-SA-Cysteamine with Mucin by the formation of the disulfide bond, and the inserted photo for the formation of hydrogel from the liquid solution of the PVA-SA-CYS and mucin.

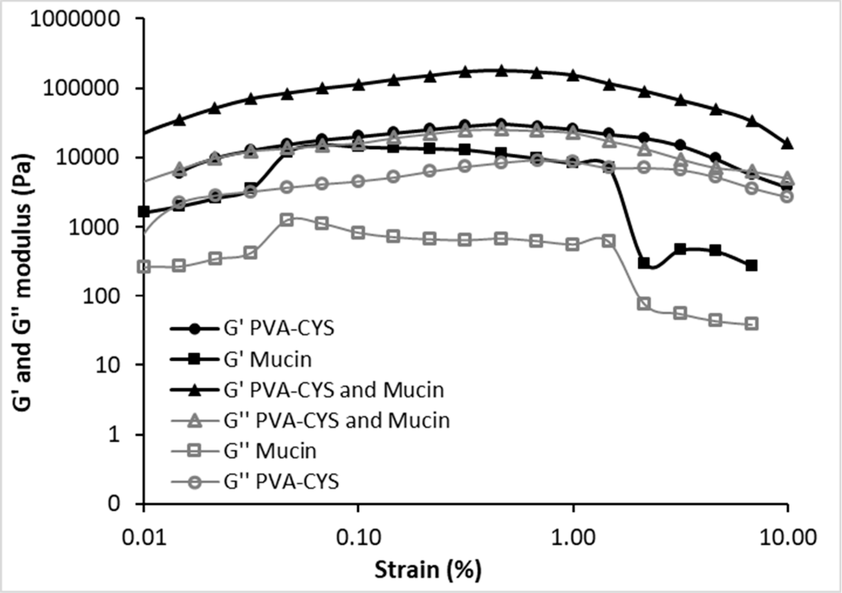

Figure S5. Storage modulus (G') and loss modulus (G'') as a function of strain (%) for the PVA-Cysteamine, mucin, and mixture

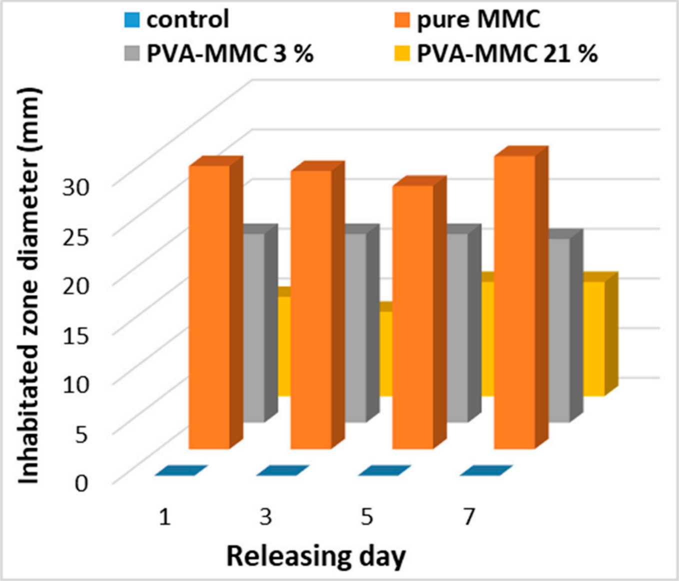

Figure S6. The obtained diameter values of the corresponding specific inhibition zone for 7 days of antibacterial effects of pure MMC and encapsulated forms on *Staphylococcus aureus (MRSA)* test bacteria.

Table S1. Interpretation of the release experiments using different kinetic models.

Sample	Zero-order model		First-order model		Higuchi model		Hixson-Crowell model		Korsmeyer-Peppas model		
	r^2	k (h^{-1})	r^2	k (h^{-1})	r^2	k ($h^{-1/2}$)	r^2	k ($h^{-1/3}$)	n	r^2	k (h^{-n})
Pure MMC	0.277	0.249	0.546	0.011	0.491	4.327	0.125	0.006	0.161	0.722	49.59
Physical mixture	0.124	0.199	0.194	0.005	0.312	3.849	0.066	0.006	0.166	0.565	46.77
PVA-MMC (3%)	0.879	0.404	0.944	0.006	0.985	5.429	0.673	0.018	0.695	0.99	2.254
PVA-MMC (10%)	0.948	0.315	0.982	0.004	0.982	4.068	0.748	0.019	0.841	0.995	1.459
PVA-MMC (21%)	0.921	0.205	0.948	0.003	0.992	2.776	0.711	0.015	0.786	0.992	1.354
